# 

**Published:** 1893-03

**Authors:** 


ESTABLISHED 1855. SUBSCRIPTION, $2.00.

### ATLANTA MEDICAL AND SURGICAL JOURNAL.

492
SURGEON GENL'S OFFICE 147498 LIBRARY.

OLD SERIES, VOL. XXVI. NEW SERIES, VOL. X. | ATLANTA, GA., MARCH, 1893. No. 1.

LUTHER B. GRANDY, M. D., MANAGING EDITOR. M. B. HUTCHINS, M. D. BUSINESS MANAGER.

